# Tilting the balance of life and death: navigating DNA replication stress in cancer therapy

**DOI:** 10.1038/s12276-026-01745-9

**Published:** 2026-06-05

**Authors:** Natalie Lo, Hyungjin Kim

**Affiliations:** 1 Department of Pharmacological Sciences, State University of New York at Stony Brook, Stony Brook, NY USA; 2 Medical Scientist Training Program, Renaissance School of Medicine at Stony Brook University, Stony Brook, NY USA; 3 Stony Brook Cancer Center, Renaissance School of Medicine at Stony Brook University, Stony Brook, NY USA

**Keywords:** Cancer therapy, DNA damage and repair

## Abstract

Genotoxic anticancer therapies exploit the heightened DNA replication stress of cancer cells. Members of the phosphoinositide-3-kinase-related kinase (PIKK) family, including ATR, ATM and DNA-PKcs, orchestrate the DNA damage response at stalled and broken replication forks, triggering complex signaling cascades that dictate cell fate. p53 plays a pivotal role in regulating the processes of cell cycle, DNA repair and programmed cell death, although p53-independent regulatory mechanisms are also evident. Genotoxic drugs often induce therapy-induced senescence, a state of dynamic and reversible cytostasis that promotes drug resistance and unfavorable patient outcomes. Conversely, severe replication stress culminates in DNA replication catastrophe, a manifestation of irreversible fork collapse with extensive single-stranded DNA accumulation that ultimately results in cell death. Therefore, the magnitude of DNA replication damage and engaged signal transduction pathways determine the outcome of genotoxic therapy. Understanding the molecular basis of the replication stress response that drives therapy-induced senescence versus cell death has far-reaching implications for enhancing the cytotoxic efficacy of anticancer regimens. Here we review the mechanisms of the DNA replication stress response, focusing on pharmacological interventions designed to shift the balance from survival to cell death.

## Mechanisms of DNA replication stress in cancer treatment outcomes

The intricate dance between DNA replication stress and cancer therapy is at the forefront of this Review. It highlights how cancer cells, under constant replication stress due to rapid proliferation and checkpoint dysfunction, exploit the DNA damage response (DDR) to survive. The authors delve into the mechanisms by which cancer cells manage replication stress, focusing on the roles of ATM, ATR and DNA-PKcs kinases in DDR signaling. They explore how these pathways influence cell fate decisions, toggling between survival strategies like senescence and death pathways such as apoptosis. Key findings include the identification of replication gaps as critical vulnerabilities in BRCA-deficient cancers, which can be therapeutically targeted with PARP inhibitors. The Review suggests that combining genotoxic therapies with senolytics or immune checkpoint inhibitors could enhance anticancer efficacy. Future research should aim to unravel the specific DDR signaling pathways across different genetic backgrounds to refine personalized cancer therapies. This summary was initially drafted using artificial intelligence, then revised and fact-checked by the author.

## Introduction

Our genome, composed of approximately 3 billion base pairs of double-stranded DNA (dsDNA), is under constant attack from endogenous and exogenous factors that compromise its integrity. These insults typically exert toxicity by damaging DNA bases, distorting the structure of DNA strands and interfering with DNA replication and transcription, thereby inducing DNA mutations, strand breakage and chromosome aberrations. The accumulation of such genetic alterations, referred to as genome instability, is a primary driver of tumor development and progression^1^. To counteract this, cells have evolved a sophisticated network of the DNA damage response (DDR) to detect specific DNA lesions and initiate repair signaling^2^. This network coordinates cell cycle checkpoints to allow time for DNA repair; however, if damage is irreparable, cells either enter permanent growth arrest known as senescence or trigger self-elimination pathways such as apoptosis and necrosis. These mechanisms constitute a critical anticancer barrier that limits oncogenic transformation^3^. Conversely, disruption of the DDR allows cells to bypass this barrier, representing a key step toward tumorigenesis. This escape is frequently mediated by mutations of *TP53*, checkpoint dysregulation or mutations in caretaker genes that ensure accurate DNA replication and repair.

The choice between survival and self-destruction following DNA damage is a programmed response; physical DNA lesions are recognized and transduced into signaling cascades that dictate cell fate^4^. Therefore, while the extent of DNA damage influences this decision, the outcome ultimately depends on the integration and propagation of signals through appropriate downstream DDR processes. Consequently, manipulating the balance between DNA repair and cell death holds important implications for maximizing the cytotoxicity of anticancer therapies. DNA replication stress, a condition that hinders the progression of DNA replication forks, is particularly relevant to oncology as cancer cells exhibit persistent replication stress due to hyperproliferation and checkpoint dysfunction^5^. Many anticancer therapies are therefore genotoxic and designed to exacerbate this stress to selectively kill cancer cells. These include cytotoxic chemotherapy, DNA replication checkpoint inhibitors and targeted therapies to inhibit DNA repair^6^. However, the ability of cancer cells to evade death, notably through induction of senescence, hampers therapeutic efficacy and drives drug resistance and disease recurrence^7^. A deeper understanding of the molecular basis governing therapy outcomes is therefore critical for developing improved therapeutic strategies. This Review focuses on the complex network of proximal and distal regulators that control cell fate decision during DNA replication stress and discusses their implications for modulating the effectiveness of genotoxic therapy.

## Conversion of DNA lesions to DDR signaling

**Fig. 1:**
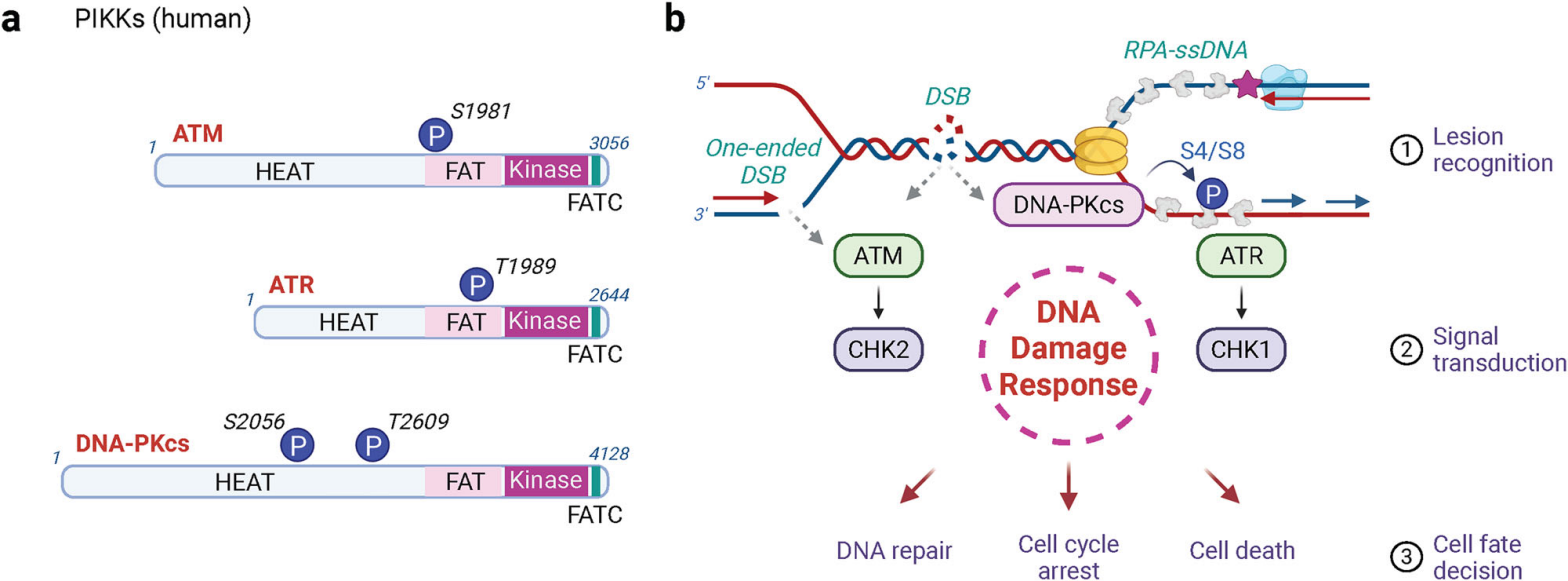
DNA damage signal transduction. **a** Domain architecture of PIKK family kinases with key autophosphorylation sites. **b** DNA lesions activate ATM–CHK2 at DSBs and ATR–CHK1 at RPA-coated ssDNA. DNA-PKcs contributes to replication stress signaling at stalled forks. The magnitude of DDR signaling coordinates DNA repair, cell cycle arrest and cell death.

The DDR is a signal transduction process through which cells detect (that is, recognition of DNA damage), transduce (that is, propagation of DNA damage signals) and respond to (that is, decision on cell fate) genotoxic insults^8^. The DDR is primarily driven by protein phosphorylation mediated by members of the phosphoinositide-3-kinase-related kinase (PIKK) family: ataxia telangiectasia mutated (ATM), ataxia telangiectasia and Rad3‑related (ATR), and DNA-dependent protein kinase (DNA‑PK; comprising the catalytic subunit DNA-PKcs and the Ku70/80 heterodimer)^9^. These kinases share a C-terminal kinase domain, which is flanked by an upstream FRAP–ATM–TRRAP (FAT) and a downstream FAT C-terminal (FATC) domain, while a variable number of α-solenoid HEAT repeats is located at the N terminus to mediate protein–protein interactions^10^ (Fig. 1a). One key feature of the DDR kinases is damage-induced autophosphorylation at S/TQ motifs. Notable sites include Thr1989 of ATR and Ser1981 of ATM, both located within the FAT domain^11^. For DNA-PKcs, a cluster of S/TQ phosphorylation sites is found in the HEAT repeats, while Ser2056 and Thr2609 are the most prominent^12^. Although the functional consequences of autophosphorylation in directly regulating DDR kinase activity remain incompletely resolved, these modifications are widely used as surrogate markers of kinase activation in response to genotoxic stress^13,14^.

Once initiated, the ATM–CHK2 and ATR–CHK1 pathways constitute core DDR signaling cascades activated by double-strand DNA breaks (DSBs) and single-stranded DNA (ssDNA), respectively (Fig. 1b). The effector kinases CHK1 and CHK2 propagate signaling to engage cell cycle checkpoints, DNA repair or apoptosis. CHK1 is indispensable for embryonic development, while CHK2 is largely dispensable^15,16^. While organisms can survive with mutations of *ATM* or other components necessary for DSB repair, such defects predispose them to cancer at the cost of genomic instability^17^. Failure to properly engage ATM signaling is associated with the acquisition of therapy resistance^18^. By contrast, the ATR–CHK1 axis plays a central role in activating the DNA replication checkpoint and is therefore essential for the survival of many cell types, including cancer cells^19^. The basal activity of ATR monitors the amount of replication protein A (RPA), an ssDNA-binding protein complex that protects ssDNA at DNA replication forks, acting as a surveillance mechanism to limit excessive ssDNA exposure and fork stalling^20^. ATR and CHK1 prevent excessive dormant origin firing by restraining cyclin-dependent kinase (CDK)-mediated phosphorylation and blocking CDC45 loading at replication origins^21^. Under replication stress, ATR permits local dormant origin firing to promote DNA synthesis while suppressing origin firing in later-replicating regions^22^. This coordination of cell cycle and replication fork stabilization ensures that cells do not enter mitosis when DNA replication is compromised. The reliance of cancer cells on the ATR–CHK1 pathway creates a therapeutic vulnerability to pharmacological inhibition of ATR and CHK1^23^. ATM loss confers increased sensitivity to ATR inhibition, supporting the notion that impaired DDR signaling in ATM-deficient cells exacerbates replication stress and potentiates fork collapse^24^.

DNA substrate specificity dictates the mode of DDR kinase activation. ATM activation in response to DSBs requires a dimer-to-monomer transition and is facilitated by binding to the C terminus of NBS1 within the MRE11–RAD50–NBS1 (MRN) complex, which recruits ATM to DNA breaks and stimulates its activity^25^. The H3K9me3 histone mark at damaged chromatin further contributes to ATM activation by recruiting the TIP60/KAT5 acetyltransferase, which acetylates ATM at the FATC motif^26^. ATM can also be activated by oxidative stress through the formation of disulfide-linked dimers, a process that occurs independently of MRN under hypoxic conditions^27^. By contrast, ATR is primarily activated by ssDNA exposed at stalled forks, arising from uncoupling of replicative helicase and polymerase activities or nucleolytic processing of DNA ends. ATR is recruited to RPA-coated ssDNA through its partner ATRIP^28^. ATR activation occurs at ssDNA/dsDNA junctions and requires its dedicated activator, TOPBP1^29^. This is further mediated by the interaction of TOPBP1 with the RAD9–RAD1–HUS1 (9–1–1) complex, which is loaded onto the RPA-coated ssDNA/dsDNA junctions by the RAD17–RFC complex^30^. While TOPBP1 predominantly mediates ATR activation during replication stress, ETAA1, an additional ATR activator that directly binds RPA, is critical for ATR activation during unperturbed S-phase replication and mitosis^31,32^. Through ETAA1-dependent signaling, ATR also restricts the S/G2 transition by suppressing transactivation of the FOXM1-dependent mitotic gene network; consequently, ATR inhibition results in premature mitotic entry with underreplicated DNA^33,34^.

DNA-PKcs is distinct in its dual roles in DSB repair and regulation of replication fork dynamics during replication stress. On the one hand, DNA-PKcs is activated at DSB ends following recruitment by the Ku70/80 heterodimer and initiates repair via nonhomologous end joining (NHEJ). Its catalytic activity is necessary for the recruitment and activation of downstream NHEJ factors, including Artemis, XRCC4 and DNA ligase IV^35^. On the other hand, DNA-PKcs localizes at replication forks and cooperates with ATR signaling during replication stress^36^. Following initial RPA32 phosphorylation at Ser33 by ATR, DNA-PKcs catalyzes subsequent phosphorylation of RPA32 at Ser4 and Ser8, an event essential for intra-S phase checkpoint activation. Cells expressing a Ser4/Ser8 phosphorylation-defective RPA32 mutant exhibit defective checkpoint activation, impaired stalled fork recovery and compromised homologous recombination (HR) repair, culminating in mitotic catastrophe and cell death^37,38^. DNA-PKcs has also been shown to form complexes with the RPA heterodimer at unperturbed replication forks^39^. Importantly, DNA-PKcs promotes stalled fork remodeling independently of NHEJ, underscoring its context-dependent role in the replication stress response^36^. Under conditions of moderate replication damage, ATR inhibition can be compensated by DNA-PKcs-dependent activation of CHK1 to stabilize stalled forks, further highlighting the role of DNA-PKcs in coordinating cellular responses to DNA replication stress^40^.

## Emerging sources of DNA replication stress

As DNA replication stress persists, cancer cells become more reliant on the replication stress response for their survival. Due to an inherent loss of DNA repair capacity, cancer cells exhibit high levels of unrepaired lesions. Replication errors and the misincorporation of ribonucleotides are also predominant sources of endogenous replication stress. Aberrant DNA secondary structures, such as hairpins and G-quadruplexes formed in DNA nucleotide repeats and fragile sites, increase vulnerability to replication stress. The replisome often collides with the transcription machinery, which results in the hybridization of nascent mRNA to DNA, thereby forming R-loops. The hyperactivation of oncogenes dysregulates origin firing and limits available nucleotide pools. Many conventional cytotoxic chemotherapies form DNA adducts, cause strand breakages and target DNA replication processes. The inhibition of topoisomerase activities accumulates replication-associated DSBs and DNA–protein adducts that ultimately cause fork collapse. In this section, we will touch upon key emerging sources of DNA replication damage that are relevant to anticancer therapy.

### Replication-associated and post-replicative ssDNA gaps

**Fig. 2:**
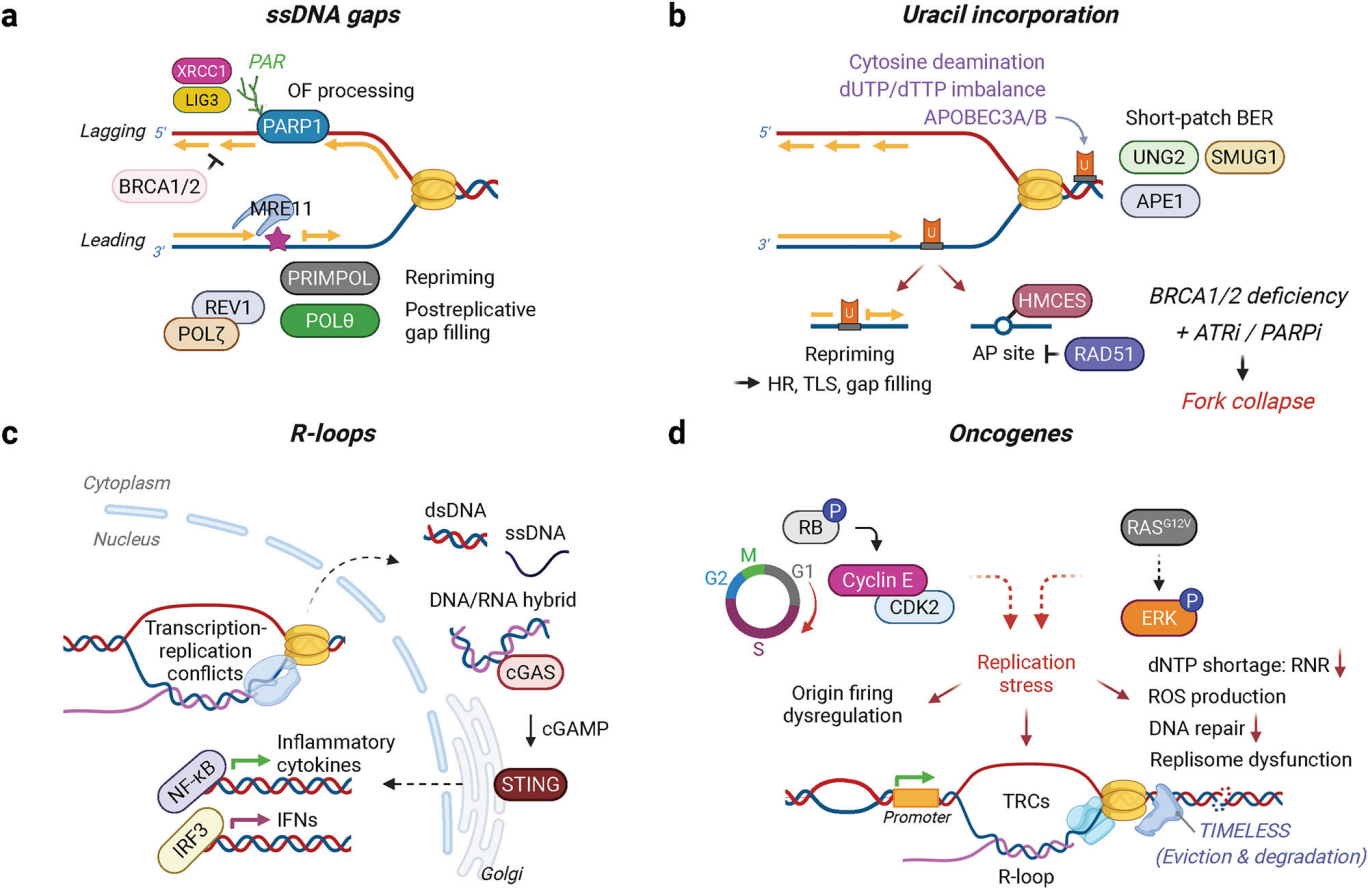
Cellular responses to DNA replication stress. **a** PARP1 inhibition disrupts Okazaki fragment processing, generating lagging strand ssDNA gaps that are exacerbated by *BRCA1*/*2* deficiency. PRIMPOL-mediated repriming generates leading strand gaps, which can be expanded by MRE11 and filled by TLS or POLθ. **b** Genomic uracil is processed by BER. At replication forks, AP incision can generate one-ended DSBs unless protected by HMCES or RAD51. BRCA1/2 loss or ATR/PARP inhibition sensitizes forks to collapse. **c** R-loops from transcription–replication conflicts (TRCs) activate cGAS–STING signaling and downstream IRF3 and NF-κB-mediated responses. **d** Oncogene activation induces replication stress through dysregulated origin firing, transcription–replication collisions, dNTP depletion, ROS accumulation, impaired DNA repair and replisome dysfunction.

Poly(ADP-ribose) polymerase (PARP) inhibitors (PARP*i*s) induce synthetic lethality in cancers harboring *BRCA1* or *BRCA2* mutations^41^. The classic DSB-centric model proposes that PARP*i*-treated cells rely on BRCA1/2-dependent HR and stalled fork protection to resolve DNA lesions arising from replication-associated one-ended DSBs, PARP trapping on DNA, and replication–transcription conflicts^42^. More recent evidence, however, identifies the accumulation of ssDNA gaps as a central vulnerability of BRCAness that dictates the therapy response to PARP*i*^43^ (Fig. 2a). Both defective lagging strand maturation and PRIMPOL (primase and DNA-directed polymerase)-dependent repriming at the leading strand contribute to the formation of ssDNA gaps. PARP activity constitutes a backup Okazaki fragment processing pathway that occurs behind replication forks to resolve ssDNA gaps formed during discontinuous lagging strand synthesis^44,45^. These gaps accumulate in BRCA1/2-deficient cells concomitantly with elevated S-phase specific PARylation, and lagging strand gaps promote fork degradation in this context^46,47^. These cells also exhibit compromised postreplicative repair of ssDNA gaps^48^. Furthermore, PARP*i* resistance conferred by loss of 53BP1 can be reversed by the deletion of LIG3, which functions in a backup PARP1-dependent Okazaki fragment maturation pathway, thereby linking BRCAness to defects in lagging strand synthesis and subsequent synergistic fork instability upon PARP inhibition^49^.

PRIMPOL generates leading strand gaps by repriming DNA synthesis downstream of lesions at stalled forks, a process elevated in BRCA1/2-deficient cells^50^. These gaps can be filled by translesion DNA synthesis (TLS), during which BRCA1/2 restricts MRE11-mediated gap expansion in the G2 phase^48^. Recent studies further identified polymerase θ (POLθ) as a mediator of postreplicative gap filling. While POLθ acts as a critical backup for DNA repair in HR deficiency via alternative-end joining (alt-EJ), POLθ also cooperates with LIG1 and FEN1 to seal ssDNA gaps, thereby preventing MRE11-dependent conversion of these gaps into DSBs^51–53^. MRE11 activity is further implicated in the generation and expansion of ssDNA gaps in BRCA1/2-deficient cells^54^. PARP*i* accelerates fork progression while leaving behind ssDNA gaps, a process that is exacerbated in BRCA1/2-deficient cells and leads to excessive ssDNA accumulation^55,56^. In addition, PARP trapping can cause ssDNA gaps to persist across cell cycles, leading to a progressive induction of DSBs and fork collapse^57^.

Whether ssDNA gaps, rather than DSBs, represent the primary determinant of the therapeutic response remains controversial. For instance, a *Brca2*^*S3214A*^ separation-of-function mutant reveals that loss of HR, rather than ssDNA gap accumulation or fork deprotection, drives tumorigenesis and chemosensitivity^58^. By contrast, studies with a RAD51^T131P^ mutant, which causes excessive nascent strand degradation in a dominant-negative fashion, suggest that DSB repair capacity alone is insufficient to predict PARP*i* sensitivity^46^. Moreover, studies show that acquired therapeutic resistance is better related to mechanisms of ssDNA gap suppression, including restrained fork progression, gain of TLS, or gap protection by RPA, while defective HR or nascent strand degradation is not directly associated with the therapeutic response^59^. Many genotoxic conditions are lethal not only in cells with BRCAness but also in those with defective XRCC1, which plays little role in HR but is involved in single-strand break repair, highlighting the importance of genotoxicity arising from unresolved gaps as a determinant of response to cancer therapy^60^. Because ssDNA gaps can be converted into DSBs during the subsequent cell cycle following PARP*i* treatment, these lesions may not be mutually exclusive. Nonetheless, accumulating evidence suggests that DSBs are not invariably lethal, whereas excessive ssDNA gap accumulation under severe replication stress is sufficient to commit cells to apoptosis^54,61^.

### Uracil-induced replication stress

Erroneous genomic uracil incorporation via cytosine deamination or imbalanced nucleotide pools is an emerging source of replication fork instability (Fig. 2b). The APOBEC family of cytidine deaminases catalyzes mutagenic uracil formation in DNA, driving C-to-T transitions and age-related mutational signatures in cancer^62^. Deamination of 5-hydroxymethyl-cytosine to 5-hydroxymethyl-uracil further generates mutagenic U:G mismatches. Uracil incorporated into nascent strands is repaired postreplicatively by short-patch base excision repair (BER), initiated by uracil DNA glycosylases UNG2 or SMUG1 and followed by incision of the resulting apurinic/apyrimidinic (AP) site by APE1^63^. The ensuing single-strand breaks are recognized and resolved by PARP1, XRCC1, Polβ and LIG1.

By contrast, uracil incorporation into the template strand near replication forks leads to AP site incision that can generate one-ended DSBs and fork collapse in the absence of HR repair. AP sites are shielded at replication forks by covalent binding of HMCES (5-hydroxymethylcytosine binding, embryonic stem cell specific)^64^. HR-deficient cells are particularly sensitive to HMCES–DNA–protein cross-links induced by 5-hydroxymethyl-uracil, leading to replication fork collapse and chromosomal aberrations^65^. Similarly, RAD51 nucleofilaments protect replication forks by preventing MRE11-dependent cleavage at AP sites^66^. Excessive accumulation of 5-hydroxymethyl 2′-deoxyuridine 5′-monophosphate due to deficiency of DNPH1, a nucleotide pool-sanitizing enzyme, underlies the synthetic lethality of PARP inhibition in BRCA-deficient cells^67^. Incorporation of 5-chloro-2′-deoxyuridine ahead of replication forks likewise induces fork collapse and hypersensitivity in BRCA-deficient contexts^68^. Furthermore, genomic uracil processing selectively kills BRCA-deficient tumors^69^. Together, these findings establish genomic uracil and unresolved BER intermediates as critical determinants of replication fork instability and PARP*i* sensitivity. Interestingly, accumulation of unprocessed genomic uracil in the absence of UNG2 slows replication fork progression and enhances PRIMPOL-mediated repriming, resulting in ssDNA gap accumulation^70^. Therefore, while uracil lesions can be tolerated independently of BER, repriming-associated gap formation renders replication forks vulnerable to collapse unless compensated by HR, TLS or gap-filling pathways^71^. Indeed, APOBEC3B-induced uracil accumulation promotes APE1-mediated DNA breakage, hyperactivation of PARP1 and increased sensitivity to ATR inhibition, highlighting uracil-induced replication stress as a vulnerability that can be therapeutically exploited^72^.

### Replication stress from R-loops—connection to inflammation

R-loops, three-stranded nucleic acid structures consisting of RNA–DNA hybrids and a displaced DNA strand, are an emerging source of replication stress arising from collisions between replication and transcription machineries^73^. Aberrant accumulation of R-loop-derived nucleic acids is sensed by pattern recognition receptors, triggering innate inflammatory signaling pathways that influence therapeutic response. The cGAS (cyclic GMP–AMP synthase)–STING (stimulator of interferon genes) pathway is particularly relevant, which detects cytoplasmic nucleic acids to activate interferon regulatory factor 3 (IRF3) and induce expression of interferon (IFN)-stimulated genes and immunomodulatory cytokines^74^ (Fig. 2c). Type I IFN production enhances antitumor immunity by promoting cytotoxic activity of CD8^+^ and CD4^+^ T cells and natural killer (NK) cells^75^. An R-loop scoring model based on single-cell RNA sequencing revealed that low R-loop burden correlates with T cell exhaustion and compromised therapeutic effects in lung adenocarcinoma^76^. Conversely, excessive R-loop accumulation and genome instability causes chronic inflammation and disrupts hematopoietic homeostasis, as observed in myelodysplastic syndrome^77^. Chronic R-loop-driven inflammation may also promote an immunosuppressive tumor microenvironment, facilitating immune evasion. Yet, this intimate link between R-loops and inflammation presents therapeutic opportunities. For instance, constitutive nuclear factor-κB (NF-κB) activation enhances R-loop formation in adult T cell leukemia, selecting for loss of transcription-coupled nucleotide excision repair endonucleases. While this adaptation permits escape from senescence, it confers hypersensitivity to ultraviolet irradiation^78^. Loss of SMARCAL1, an SNF2-family DNA translocase, enhances antitumor immunity through R-loop accumulation, activation of cGAS–STING signaling and downregulation of PD-L1 expression, substantiating R-loop modulation as a target for cancer immunotherapy^79^.

### Oncogene-induced replication stress

Oncogene-induced replication stress is a major driver of genome instability during cancer initiation and progression. Active oncogenes disrupt replication fidelity and kinetics, causing accumulation of S phase-specific DNA damage that triggers oncogene-induced senescence (OIS), an intrinsic barrier to tumorigenesis. Accordingly, disruption of DDR pathways enables cells to bypass OIS and gain unrestrained proliferative capacity, fueling cellular transformation^80^. Oncogene-induced replication stress arises from multiple mechanisms but is predominantly linked to altered DNA replication dynamics and cell cycle dysregulation (Fig. 2d).

Oncogenic RAS and Cyclin E expression generates distinct DNA fragility landscapes that frequently overlap with cancer-associated breakpoints^81^. These oncogenes promote DNA hyperreplication through increased origin firing and transcription, leading to transcription–replication conflicts and R-loop accumulation^82^. MYC affects replication origin activity by dysregulating CDK and E2F expression, reducing origin firing under some conditions while inducing excessive origin firing when overexpressed^83^. Defective nucleotide metabolism and elevated reactive oxygen species (ROS) further contribute to oncogene-associated replication stress. RAS downregulates ribonucleotide reductase subunit M2 (RRM2), reducing dNTP levels and impairing DNA synthesis^84^. It also compromises DSB repair by downregulating BRIP1, causing dissociation of its binding partner BRCA1 from chromatin^85^. Furthermore, RAS induces replication fork instability by promoting degradation of TIMELESS (TIM), a core replisome component, thereby enforcing OIS^86^. Disengagement of TIM from the replisome is also exacerbated by ROS accumulation during oncogene activation^87^.

High-grade serous ovarian cancer (HGSOC) exemplifies a malignancy driven by high copy number alterations and chromosomal instability, and is therefore considered a model of elevated replication stress^88^. In addition to frequent HR deficiency caused by *BRCA1*/*2* mutations, HGSOC is characterized by nearly universal pathogenic *TP53* mutations and amplification of *CCNE1* (Cyclin E1) in approximately 20% of cases^89^. *MYC* amplification is also prevalent in HGSOC. Cyclin E1 drives transformation of fallopian tube secretory cells, the presumed cell of origin for HGSOC^90^. Accordingly, selective CDK2 inhibition targets Cyclin E1-overexpressing HGSOC by inducing cell cycle arrest and senescence, exploiting oncogene-induced replication stress for therapy^91^.

## Fate decision under DNA replication stress

### Survival strategies

**Fig. 3:**
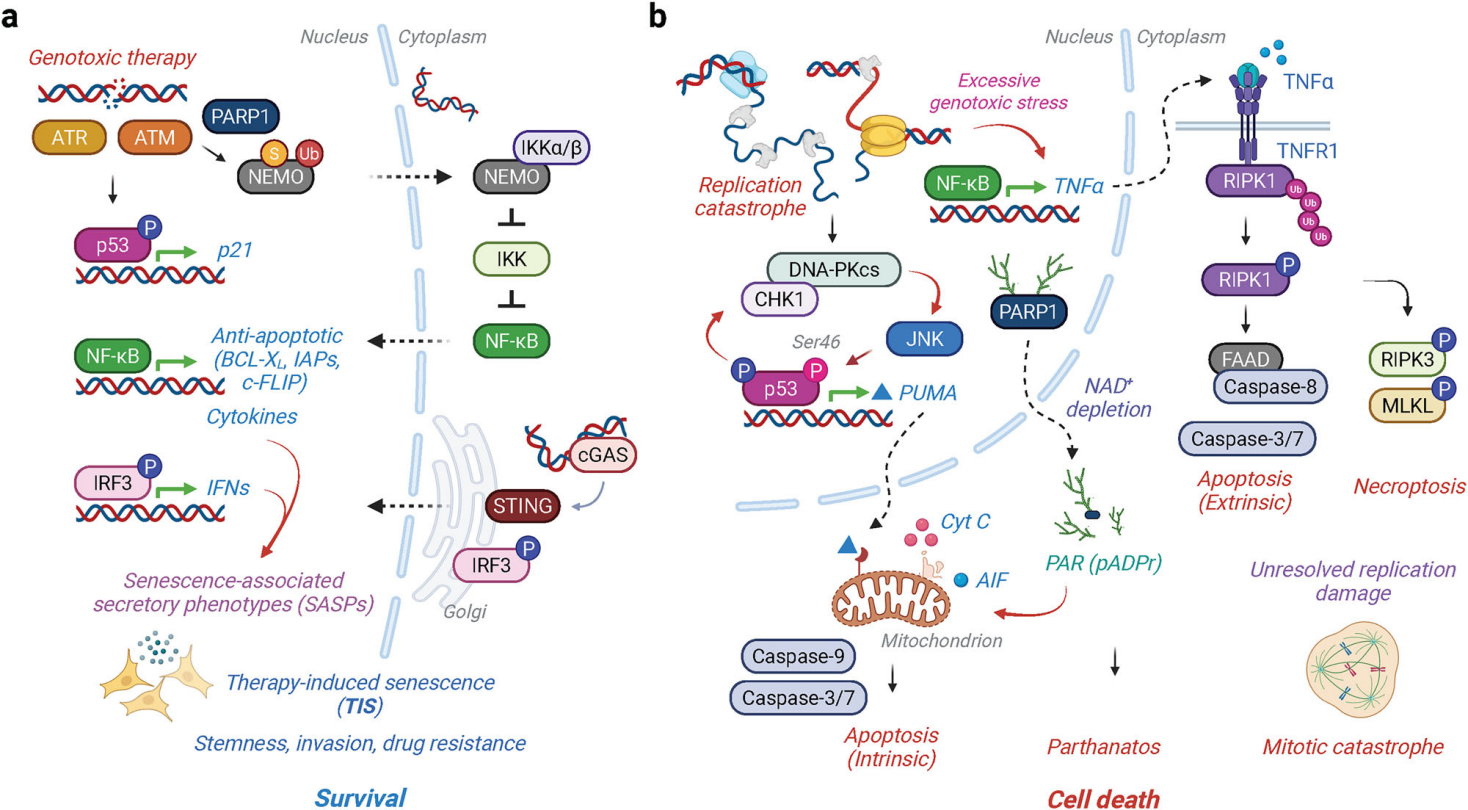
Cell fate decision under genotoxic stress. **a** Genotoxic therapies activate ATM/ATR to induce p53-dependent cell cycle arrest as well as NF-κB and cGAS–STING signaling, establishing TIS characterized by antiapoptotic signaling and SASP-driven inflammation. **b** Excessive replication stress causes ssDNA accumulation and replication catastrophe, triggering p53-dependent apoptosis. Other cell death mechanisms include TNFα–RIPK1-mediated apoptosis or necroptosis, PARP1-driven parthanatos or mitotic catastrophe due to unresolved replication damage.

Cells experiencing replication stress undergo senescence, a state of cell cycle arrest that maintains a nonproliferative yet metabolically active phenotype. While progressive telomere attrition is sensed as persistent DNA damage sufficient to trigger replicative senescence and limit genome instability, a variety of endogenous genotoxic stresses, including oncogene activation, oxidative stress and nucleotide depletion, activate signaling cascades that enforce cell cycle withdrawal (Fig. 3a). This is primarily mediated by the DNA damage-induced p53–p21^CDKN1A^ and the mitogenic stress-activated p16^INK4a^–RB pathways, which converge to inhibit CDKs required for cell cycle progression. Senescence thus acts as an intrinsic barrier to tumorigenesis, and senescent cells are commonly detected in premalignant lesions of patients with cancer.

Conventional anticancer therapeutics, including cytotoxic chemotherapy or radiotherapy, induce DNA damage sufficient to trigger senescence through activation of the ATM and ATR pathways. This phenomenon, termed therapy-induced senescence (TIS), is considered an antitumor mechanism by preventing cancer cell proliferation^92^. A hallmark of senescence is the production and secretion of pro-inflammatory cytokines and immunomodulatory factors, collectively known as the senescence-associated secretory phenotype (SASP)^93^. IL-1α, a key SASP cytokine, promotes antitumor immunity by transcriptionally upregulating IL-6 and IL-8, together enhancing immune surveillance mediated by macrophages, NK cells and cytotoxic T lymphocytes, as demonstrated in mouse models of lung, liver and breast cancer^94^. While restoration of p53 activity is critical for reinforcing growth arrest and facilitating immune clearance, paracrine SASP signaling to surrounding nonsenescent tumor cells can further suppress tumor progression^95^.

However, accumulating evidence indicates that TIS is not an irreversible fate; instead, persistent TIS enables cancer cells to evade therapeutic cytotoxicity and repopulate tumors with increased aggressiveness. This does not necessarily indicate the actual reversion of tumor cells to their pre-senescence status; rather, the escape from a transient ‘senescence-like state’ is accompanied by dynamic genetic and metabolic changes that contribute to a more malignant phenotype^96^. For instance, a recent genomic analysis of breast cancer cells undergoing therapy reveals unique transcriptome profiles distinct from those of parental and repopulating cells, highlighting the transient nature of TIS^97^. During this process, SASP factors exert protumorigenic effects by supporting a chronic pro-inflammatory microenvironment that enhances tumor growth. IL-6-mediated recruitment of myeloid-derived suppressor cells establishes an immunosuppressive niche by inhibiting CD8^+^ T and NK cells^98^. Secretion of matrix metalloproteinases and vascular endothelial growth factors from senescent cells promotes metastasis and angiogenesis^99^. TIS is also associated with the acquisition of stem-like properties and increases the likelihood that residual cells re-enter the cell cycle^100^. Therefore, these features enable TIS to function as a survival strategy for tumors, supporting a transient state of dormancy that ultimately contributes to disease recurrence and poor patient outcomes.

The NF-κB pathway is a major inducer of senescence and is directly regulated by ATM under genotoxic stress^101^. Canonical NF-κB signaling is initiated by inflammatory stimuli engaging cell surface receptors, leading to the activation of the IκB kinase (IKK) complex, composed of two catalytic subunits (IKKα and IKKβ) and a regulatory subunit, NF-κB essential modifier (NEMO/IKKγ)^102^. Activated IKK phosphorylates and degrades the inhibitory protein IκBα, allowing NF-κB to translocate into the nucleus and transcriptionally induce prosenescence genes. Notably, ATM-mediated posttranslational modification of NEMO provides a critical link between nuclear DDR signaling to cytoplasmic NF-κB activation^103^. This process involves ATM-mediated phosphorylation of NEMO at Ser85, followed by SUMOylation and mono-ubiquitination at Lys277 and Lys309, which promotes nuclear export of NEMO and downstream NF-κB activation^104,105^. PARP1 functions as an essential upstream regulator of NEMO SUMOylation, thereby facilitating ATM-dependent NF-κB signaling^106^. Constitutive NF-κB activation is observed in many cancers and is associated with SASP induction, chronic inflammation, and therapy resistance^107^. Recent evidence further indicates that NF-κB supports cGAS–STING signaling by promoting STING stability, suggesting that NF-κB and cGAS–STING pathways may cooperate to establish TIS independently of p53-mediated cell cycle arrest^108^.

In addition to promoting senescence, NF-κB signaling elevates the apoptotic threshold by inducing expression of prosurvival genes. Beyond transcriptional upregulation of *BCL2L1* (BCL-X_L_), a key inhibitor of mitochondrial apoptosis, NF-κB induces antiapoptotic effectors including survivin, cellular inhibitors of apoptosis (cIAPs), and X-linked inhibitor of apoptosis (XIAP), which suppress caspase activation^109^. NF-κB also drives expression of cellular FLICE (FADD-like IL-1β-converting enzyme)-inhibitory protein (c-FLIP), which heterodimerizes with caspase-8 and prevents its activation, thereby suppressing death receptor-mediated and TNFα-induced apoptosis^110^. c-FLIP is frequently overexpressed in cancer and represents a key determinant of therapy resistance^111^. Accordingly, targeting ATM-dependent NF-κB signaling suppresses c-FLIP induction and sensitizes colorectal cancer cells to topoisomerase inhibitors through enhanced apoptosis^112^.

The AKT kinase also suppresses apoptosis by phosphorylating pro-apoptotic proteins such as BAD, thereby blocking mitochondrial apoptosis, and apoptosis signal-regulating kinase 1 (ASK1), preventing activation of c-Jun N-terminal kinase (JNK), which otherwise promotes apoptosis under genotoxic stress^113,114^. AKT further contributes to NF-κB activation by phosphorylating the IKK complex and promoting IκB degradation^115^. AKT is directly activated by DNA damage through PIKKs, and nuclear AKT enhances DDR signaling and DSB repair^116,117^. Together, prosenescence and prosurvival pathways enable cancer cells to evade genotoxic therapy and increase the risk of disease relapse.

### Death pathways

Maximizing genotoxic stress by modulating replication stress signaling can shift the balance from survival to death. When the replication checkpoint fails to adequately counteract replication stress, cells undergo DNA replication catastrophe, characterized by extensive ssDNA exposure that exhausts the nuclear pool of RPA, resulting in unprotected ssDNA and fork collapse^118^ (Fig. 3b). DDR signaling initiated by excessive replication damage can overcome the apoptotic threshold imposed by TIS, including elevated expression of antiapoptotic proteins. We previously modeled p53-dependent signaling pathways underlying replication catastrophe induced by combined replisome dysfunction and ATR inhibition^54,119^. Failure of ATR to restrain replisome activity and suppress origin firing exacerbates ssDNA accumulation caused by replisome uncoupling, culminating in apoptosis. Replication catastrophe is marked by pan-nuclear γH2AX, a biomarker for lethal replication stress and therapeutic efficacy^120^. This process is initiated by ssDNA gaps processed by MRE11, which activates a death pathway involving DNA-PKcs and CHK1^54^.

Hence, cell fate is dictated by a shift between prosurvival and prodeath functions of PIKKs that reprogram DDR signaling. Alterations in p53-dependent transcriptional outputs are central to this transition. Following phosphorylation of p53 at Ser15 and Ser20, which stabilizes the protein, additional phosphorylation at Ser46 under conditions of irreparable damage promotes apoptotic p53 function by selectively activating promoters of pro-apoptotic genes, including *PUMA*, *NOXA*, *TP53AIP1* and *PIG3*^121^. JNK is frequently coactivated by stress stimuli and phosphorylates p53 to further channel its activity toward apoptosis^122^. Homeodomain-interacting protein kinase 2 (HIPK2), in complex with p53AIP1, facilitates Ser46 phosphorylation of p53^123^; ATM/ATR-mediated stabilization of HIPK2 through disruption of its interaction with the SIAH1 ubiquitin E3 ligase provides a mechanism by which the intensity of the DDR modulates p53-driven apoptosis^124^.

In cancers harboring p53 mutations, alternative death pathways often dominate. Under excessive DNA damage, autocrine TNFα–TNFR1 feed-forward signaling promotes caspase-8 activation, supported by sustained NF-κB activity involving ATM, receptor-interacting serine/threonine-protein kinase 1 (RIPK1) and NEMO^125^. IL-8 secretion further contributes to this process, illustrating how inflammatory signaling influences cell fate decisions following DNA damage. Posttranslational modification of RIPK1 during NF-κB–TNFα signaling governs a switch from caspase-8-dependent apoptosis to regulated necrosis, or necroptosis. This pathway bypasses p53 signaling and overcomes antiapoptotic barriers (for example, IAPs or c-FLIP) by inducing MLKL-mediated membrane permeabilization and osmotic collapse^126^. Excessive PARP activation under severe genomic stress and oxidative damage depletes cellular NAD^+^, leading to mitochondrial dysfunction and parthanatos, a distinct form of regulated necrosis^127^. Extensive PAR polymer synthesis promotes release and nuclear translocation of apoptosis-inducing factor, resulting in large-scale DNA fragmentation and cell death^128^.

Mitotic failure represents a delayed cell death response in p53-deficient cancer cells resistant to genotoxic therapy. Mitotic catastrophe originates from mitotic entry following irreparable S-phase DNA damage in the context of defective G1 and G2 checkpoints, resulting in chromosome missegregation and cell death via apoptosis or necrosis^129^. This process often involves failure of cell cycle surveillance mechanisms, including the spindle assembly checkpoint, leading to prolonged mitotic arrest. Mitotic catastrophe therefore functions as both a tumor-suppressive mechanism and therapeutic vulnerability. PARP inhibition in HR-deficient cancer cells induces mitotic DNA damage characterized by chromosome bridges, lagging chromosomes and multinucleation, leading to cell death^130^. Similar outcomes are observed in MYCN-amplified neuroblastoma treated with PARP*i*^131^. The ATR checkpoint is essential for preventing mitotic catastrophe; accordingly, ATR inhibition promotes premature mitotic entry following delayed replication induced by CDC7 inhibition^132^. Combined ATR and PARP inhibition in HR-deficient cells synergistically drives mitotic failure, underscoring the therapeutic potential of enforcing catastrophic mitotic collapse to maximize genotoxic efficacy and determine cancer cell fate^133^.

## Application of cell fate decision in cancer therapy

### One-two punch therapy

**Fig. 4:**
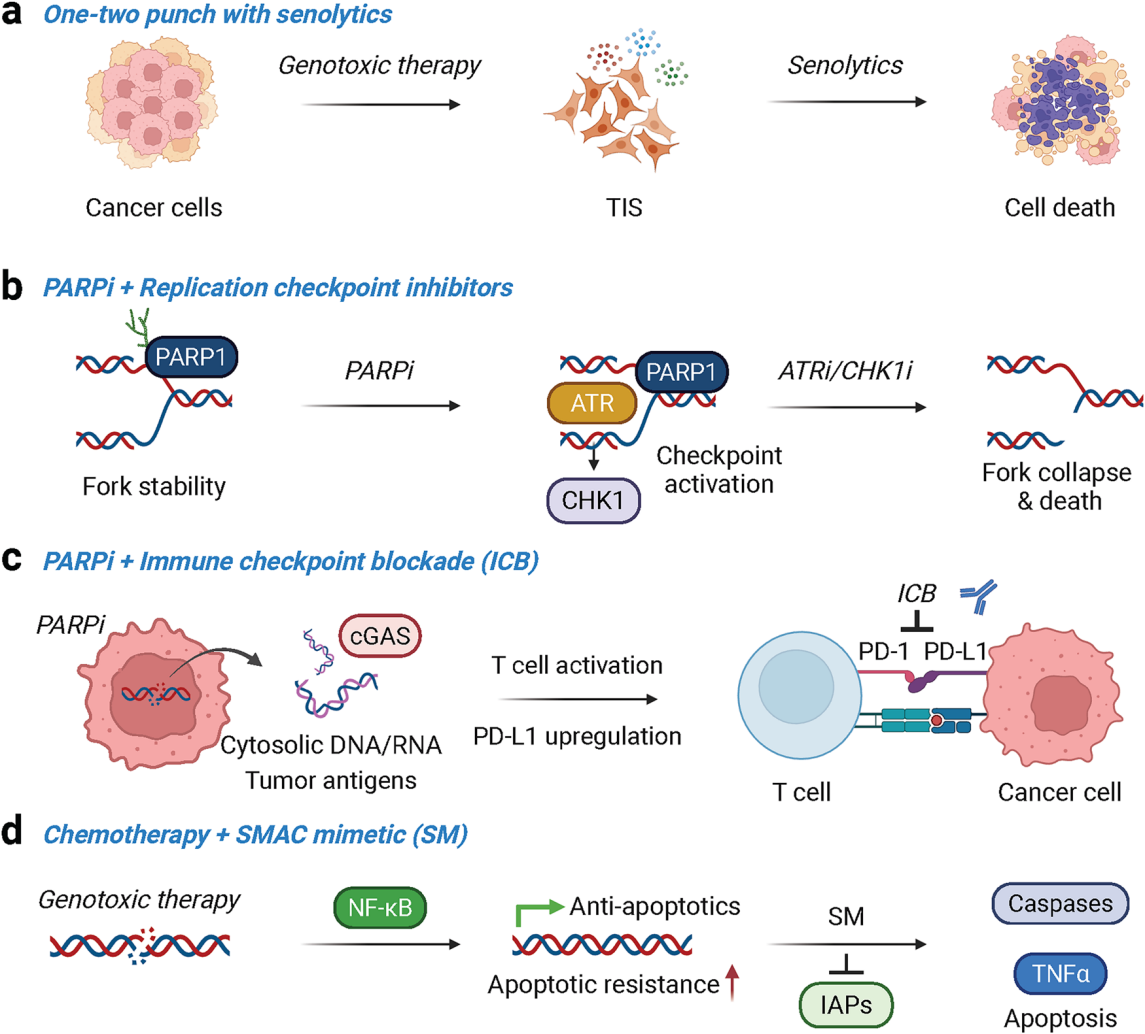
Underlying basis of combination therapies. **a** The one–two punch approach utilizes senolytic agents that can specifically eliminate therapy-induced senescent cancer cells. **b** PARP inhibition induces replication stress and activates the ATR–CHK1 checkpoint, rendering cancer cells vulnerable to replication checkpoint inhibition. **c** PARP*i* increases neoantigen production and stimulates innate immune signaling, triggering immune cell recruitment and activation, which makes cancer cells susceptible to PD-1/PD-L1 blockade. **d** NF-κB activation in response to genotoxic stress elevates the apoptotic resistance of cancer cells. SMs promote cell death by antagonizing IAPs, which are inhibitors of caspase activation, and by triggering TNFα-dependent extrinsic apoptosis.

TIS presents a unique opportunity to eliminate cancer cells through a ‘one–two punch approach’, in which the first punch induces senescence through genotoxic chemotherapy, while the second utilizes senolytics to selectively eliminate these cells via apoptosis (Fig. 4a). Preclinical models have explored the combination of genotoxic therapy with BH3 mimetics (for example, navitoclax/ABT-263 and venetoclax/ABT-199), which target antiapoptotic proteins (for example, BCL-2, BCL-X_L_ and BCL-W) that are often upregulated in senescent cells^134^. Navitoclax has been shown to selectively clear senescent cells induced by topoisomerase inhibitors^135–137^, gemcitabine^138^ and temozolomide^139–141^ in both in vitro and in vivo cancer models. Preclinical studies across various cell lines and breast cancer xenograft mouse models demonstrated that PARP*i* monotherapy or PARP*i* combined with radiotherapy induces senescence, and that subsequent treatment with ABT-263 or ABT-199 triggers cell death and suppresses tumor growth^142–146^. These findings underscore the therapeutic advantage of combining chemotherapy with senolytics to enhance therapeutic efficacy and potentially mitigate disease recurrence and relapse.

Navitoclax has advanced through multiple phase 1 clinical trials in combination with genotoxic therapies, including olaparib (NCT05358639), etoposide/cisplatin (NCT00878449), paclitaxel (NCT00891605), docetaxel (NCT00888108), gemcitabine (NCT00887757) and irinotecan (NCT01009073) (Table 1). Although navitoclax in combination with chemotherapy exhibited antitumor activity, it was poorly tolerated in several studies, leading to trial termination due to hematologic toxicities, with thrombocytopenia and anemia being the most common adverse side effects caused by on-target BCL-X_L_ inhibition^147^. This toxicity was also observed in the preclinical studies of navitoclax and ABT-737^148,149^. This challenge prompted the development of venetoclax, a selective BCL-2 inhibitor, to spare platelets. While venetoclax alleviates hematologic side effects, its senolytic efficacy is variable across different cancer types, presumably due to the requirement for BCL-X_L_ inhibition to effectively induce apoptosis^149–151^. To overcome these limitations, alternative drug-delivery strategies have been developed to selectively target senescent cells while minimizing platelet toxicity. One such strategy is the development of the prodrug Nav-Gal, a navitoclax–galactose conjugate specifically cleaved by elevated β-galactosidase activity in senescent cells^152,153^. Using proteolysis-targeting chimeras, another study converted navitoclax into PZ15227, which recruits BCL-X_L_ to the cereblon ubiquitin E3 ligase for proteasomal degradation^154^. Together, these approaches present promising strategies to overcome the clinical challenges associated with senolytic-based one–two punch therapies.

**Table 1:** List of clinical trials using genotoxic therapies.

Agent	Phase	Study identifier	Drug combination	Target malignancy	Status
**One-two punch therapy with senolytics**					
Navitoclax	Phase 1	NCT00891605	Paclitaxel	Advanced solid tumors	Completed
Navitoclax	Phase 1	NCT00888108	Doxetaxel	Advanced solid tumors	Completed
Navitoclax	Phase 1	NCT00878449	Etoposide/cisplatin	Small-cell lung cancer	Completed
Navitoclax	Phase 1	NCT01009073	Irinotecan	Advanced solid tumors	Completed
Navitoclax	Phase 1	NCT00887757	Gemcitabine	Advanced solid tumors	Completed
Navitoclax	Phase 1	NCT05358639	Olaparib	HGSOC and TNBC	Active
**PARP inhibitor with checkpoint inhibitors**					
Olaparib (ref. ^163^)	Phase 2	NCT02576444	Ceralasertib	Advanced solid tumors	Terminated
Olaparib (ref. ^164^)	Phase 2	NCT03182634	Ceralasertib	Advanced breast cancer	Unknown
Olaparib (ref. ^165^)	Phase 2	NCT03462342	Ceralasertib	Recurrent ovarian cancer	Completed
Olaparib (ref. ^166^)	Phase 1	NCT03057145	Prexasertib	Advanced solid tumors + HGSOC	Completed
**PARP inhibitor with immune checkpoint blockers**					
Niraparib (refs. ^175,176^)	Phase 1/2	NCT02657889	Pembrolizumab	TNBC or Ovarian cancer	Completed
Olaparib (ref. ^177^)	Phase 1/2	NCT02734004	Durvalumab	Advanced solid tumors	Active
Niraparib (ref. ^178^)	Phase 1/2	NCT03404960	Ipilimumab or nivolumab	Pancreatic adenocarcinoma	Completed
**Monovalent SMs**					
Xevinapant (ref. ^184^)	Phase 1/2	NCT02022098	Cisplatin, radiotherapy	Locally advanced/ untreated head and neck squamous cell carcinoma	Completed
Xevinapant	Phase 3	NCT04459715	Cisplatin, radiotherapy	Locally advanced head and neck squamous cell carcinoma	Terminated
Xevinapant	Phase 3	NCT05930938	Cetuximab, radiotherapy	Squamous cell carcinoma of head and neck	Terminated
**Bivalent SMs**					
Birinapant (ref. ^185^)	Phase 2	NCT01681368		Advanced ovarian, fallopian tube and peritoneal cancer	Terminated
Birinapant	Phase 1/2	NCT02587962	Pembrolizumab	Relapsed or refractory solid tumors	Terminated
Birinapant	Phase 1/2	NCT01486784		AML, myelodysplastic syndrome, ALL	Terminated

ALL, acute lymphoblastic leukemia; AML, acute myeloid leukemia.

### Combination therapies of PARP*i* with DNA replication checkpoint inhibitors

While PARP*i* has been successful as maintenance therapies for germline and somatic *BRCA1*/*2*-mutated cancers, acquired drug resistance remains a major clinical challenge^155^. PARP*i* induces activation of the ATR–CHK1 checkpoint, creating an opportunity for rational combination strategies to overcome resistance (Fig. 4b). Several preclinical studies in ovarian cancer models demonstrate that ATR or CHK1 inhibition resensitizes tumors to PARP*i*, resulting in increased DNA damage and cell death^155–160^. To deliver combined PARP*i* and ATR inhibitor (ATR*i*) treatment as a single entity, Gao et al. designed a dual ATR/PARP inhibitor by chemically linking ceralasertib (AZD6738) to olaparib. This fusion compound induces G2/M cell cycle arrest and elevates γH2AX levels, leading to apoptosis in triple-negative breast cancer (TNBC) cells and suppression of tumor growth in TNBC xenograft models^161^. In addition, targeting ATR downstream signaling with SRA737, a CHK1 inhibitor (CHK1*i*), resensitizes PARP*i*-resistant ovarian cancer cell lines and patient-derived xenograft models^162^.

Building on these promising preclinical studies, several phase 1 and 2 clinical trials have evaluated the combination of PARP*i* (for example, olaparib, niraparib, talazoparib and veliparib) and ATR*i* (for example, ceralasertib/AZD6738, tuvusertib/M1774, gartisertib/VX-803 and berzosertib/VE-822) in solid tumors. These trials in breast and ovarian cancers suggest that therapeutic efficacy is largely influenced by genetic context. The OLAPACO trial (NCT02576444) demonstrated a modest overall objective response rate (ORR) of 8.3%; however, patients with *ATM* mutations exhibited an ORR of 20%, and patients with HGSOC harboring *BRCA1*/*2* mutations and experiencing acquired PARP*i* resistance showed an ORR of 14%^163^. The plasmaMATCH trial (NCT03182634) reported an ORR of 23.1% in TNBC patients with germline or somatic *BRCA1*/*2* mutations^164^. The phase 2 CAPRI trial (NCT03462342) further supports the utility of this combination, reporting a 50% ORR in patients with HR deficiency^165^. Similarly, a phase 1 clinical trial combining prexasertib (CHK1*i*) with olaparib (NCT03057145) demonstrated antitumor activity and resensitization in patients with acquired PARP*i*-resistant, BRCA-deficient HGSOC (4 out of 18)^166^. Together, these studies highlight the therapeutic promise of targeting DNA replication checkpoints to overcome PARP*i* resistance.

### PARP*i* and immune checkpoint blockade (ICB)

PARP*i*-induced ssDNA gaps, particularly in BRCA1/2-deficient cells, lead to DSBs, chromosome bridges and micronucleus formation^167^. These unresolved DNA lesions result in the leakage and accumulation of DNA and RNA fragments in the cytosol, which activates the cGAS–STING pathway and stimulates antitumor immunity through enhanced cytotoxic T cell infiltration^168–171^. ICB can further enhance therapeutic efficacy by relieving inhibitory signals on T cells, particularly by targeting PD-1/PD-L1 (Fig. 4c). PARP*i*s have also been shown to upregulate PD-L1 expression in breast and lung cancer cell lines, rendering them vulnerable to disruption of the PD-1/PD-L1 axis^168,172,173^. Multiple preclinical studies show that PARP*i* combined with ICBs targeting PD-L1 (for example, pembrolizumab and nivolumab)^172,173^, PD-1 (for example, atezolizumab, durvalumab, avelumab and dostarlimab) or CTLA-4 (for example, ipilimumab and tremelimumab)^174^ induces synergistic antitumor activity in in vitro and in vivo models. Early-phase clinical trials evaluating PARP*i* and ICB combinations have yielded encouraging results in advanced solid tumors. For instance, niraparib plus pembrolizumab (NCT02657889) demonstrated antitumor activity in platinum-resistant ovarian cancer and TNBC^175,176^. The MEDIOLA trial (NCT02734004) reported promising activity of durvalumab combined with olaparib in patients with metastatic breast cancer with germline *BRCA1*/*2* mutations^177^. Niraparib combined with anti-CTLA-4 therapy (NCT03404960) showed superior progression-free survival compared with nivolumab (anti-PD-1) in platinum-sensitive pancreatic cancers^178^. Together, these studies highlight the potential of PARP*i* and ICB combinations to enhance durable responses and overcome resistance.

### Targeting antiapoptotic regulators with SMAC mimetics (SMs)

SMAC/DIABLO is a pro-apoptotic mitochondrial protein that is released into the cytosol, where it binds and degrades IAPs to enable caspase activation and the execution of apoptosis. IAPs are often upregulated in cancer, and SMs are designed to mimic endogenous SMAC proteins to counteract this apoptotic resistance^179^ (Fig. 4d). These agents can be monovalent (for example, LC161, AT-406/xevinapant/debio 1143 and GDC-0152) or bivalent (for example, birinapant/TL-32711, HGS-1029 and APG-1387/SM-1387), with bivalent compounds generally exhibiting greater potency due to their multivalent engagement of IAPs. Preclinical studies have demonstrated synergistic cell death when SMs are combined with chemotherapeutic agents^180–182^. This synergy often requires an autocrine TNFα loop to trigger the extrinsic apoptotic pathway by targeting cIAP1/2 in the death receptor complex^183^. So far, dozens of clinical trials have evaluated SMs as monotherapies or in combination with immunotherapy, genotoxic agents and radiotherapy. Phase 1 and 2 trials of combining xevinapant with chemoradiotherapy in head and neck squamous cell carcinomas (NCT02022098) reported a substantial improvement in progression-free survival^184^. However, subsequent phase 3 trials (NCT04459715 and NCT05930938) were terminated due to insufficient clinical benefit. Similarly, multiple phase 2 trials evaluating birinapant (NCT01681368, NCT02587962 and NCT01486784) were eventually terminated due to limited efficacy^185^. Overall, although SMs are considered a powerful death sensitizer, further studies are required to identify optimal combination partners and therapeutic contexts to fully unleash its apoptotic potential while minimizing toxicity and resistance.

## Conclusions and perspectives

In this Review, we discussed the origins of replication stress in cancer cells and cellular responses that dictate cell fate decisions involving survival and death. Signal transduction mediated by ATM, ATR and DNA-PKcs in response to replication damage and the resulting transcriptional programs have profound implications for tumorigenesis and therapy response. Under genotoxic therapy, cancer cells frequently exploit TIS as a survival strategy to elevate the apoptotic threshold and generate protumorigenic inflammatory signaling. The extent of p53 activation, regulated by DDR kinases, is a key determinant of pathway choice, while p53-independent mechanisms, including NF-κB- and cGAS–STING-mediated signaling, also play critical roles. Strategies aimed at maximizing genotoxic stress beyond the survival threshold, such as combining genotoxic therapies with pro-apoptotic senolytics, may enhance anticancer efficacy, provided that tumor-selective sensitization is achieved while sparing normal tissues. Addressing the fundamental question of how distinct DNA lesions are transduced to specific replication stress signaling across diverse genetic backgrounds and identifying the mediators that govern the pathway choice in a cancer-specific manner represents an important direction for future research. Ultimately, an integrated understanding of DNA repair, senescence and cell death networks will facilitate our effort to exploit DNA replication vulnerability of cancer cells for personalized anticancer therapy.
